# Molecular epidemiology of avian influenza viruses and avian coronaviruses in environmental samples from migratory bird inhabitants in Bangladesh

**DOI:** 10.3389/fvets.2024.1446577

**Published:** 2024-10-07

**Authors:** Most. Nahida Khatun, Shadia Tasnim, Md. Riabbel Hossain, Md. Ziaur Rahman, Md. Tofazzal Hossain, Emdadul Haque Chowdhury, Rokshana Parvin

**Affiliations:** ^1^Department of Pathology, Faculty of Veterinary Science, Bangladesh Agricultural University, Mymensingh, Bangladesh; ^2^Molecular Radiobiology and Biodosimetry Division, Institute of Food and Radiation Biology, Atomic Energy Research Establishment, Dhaka, Bangladesh; ^3^Department of Microbiology and Hygiene, Faculty of Veterinary Science, Bangladesh Agricultural University, Mymensingh, Bangladesh

**Keywords:** molecular epidemiology, migratory birds, AIV H4N2, AIV H4N6, avian coronavirus, phylogenetic origin

## Abstract

Migratory birds are a natural reservoir for major respiratory viruses such as the avian influenza virus (AIV) and the avian coronavirus (AvCoV). Transmission of these viruses from migratory birds to domestic birds increases the prevalence of those diseases that cause severe economic and public health concerns in Bangladesh. The study focused on active surveillance of major respiratory viral pathogens in migratory birds, molecular identification of the viruses, and their phylogenetic origin. To conduct this study, 850 environmental samples (830 fecal samples, 10 soil samples, and 10 water samples) were collected during three consecutive winter seasons from three divisions (Dhaka, Sylhet, and Mymensingh) and pooled according to the year of collection and locations, resulting in a total of 184 tested samples. Using gene-specific primers and probes in TaqMan-and SYBR Green-based RT-qPCR assays, the samples were screened for AIV and AvCoV, respectively. Out of the 184 pooled samples, 37 were found to be positive for these respiratory pathogens. Furthermore, out of the 37 (20.11%) positive respiratory pathogens, 11.96% were AIV (*n* = 22) and 8.15% were AvCoV (*n* = 15). For the first time in Bangladesh, AIV H4N2, H4N6, and AvCoVs have been found in fecal samples from migratory birds through surveillance. Phylogenetic analyses of the HA and NA genes of AIV and the polymerase gene (Orf 1) of AvCoV revealed that these strains share a close phylogenetic relationship with the isolates from wild birds in Europe and Asia. The Bangladeshi strains with Eurasian ancestry might pose a significant threat to migratory birds flying through the Asian flyways. They might also be a potential source of virus introduction and spread to poultry raised on land. These findings emphasize the significance of ongoing AIV and AvCoV surveillance in migratory birds in Bangladesh.

## 1 Introduction

The most common and major respiratory viral diseases of commercial poultry are avian influenza (AI) and avian coronavirus infection. They originated mostly from their reservoir hosts, wild aquatic birds. Migratory birds carry zoonotic and infectious pathogens all over the world. They intermingle with domestic waterfowl at their temporary residence. As a result, there is a complicated environment with the potential for pathogen exchange and host–pathogen interaction. As domestic waterfowl and migratory birds share the same water sources, there is a great chance to spread the pathogens (1). Evidence of respiratory viral pathogens, such as avian influenza viruses (AIVs) and avian gammacoronaviruses (AvCoVs), has been detected in both domestic and wild bird populations worldwide (2, 3) and in Bangladesh (1, 4).

Avian influenza (AI) is a highly contagious viral disease that affects various avian species. Influenza A viruses (IAVs) of all18 hemagglutinin (H) and 11 neuraminidase (N) subtypes, in numerous combinations, have been isolated from wild aquatic birds (5, 6). Among those IAVs, subtype H4 has been reported in avian species worldwide, including chickens, ducks, turkeys, shore birds, and wild birds, thus becoming one of the most prevalent subtypes found in wild birds in North America, Europe, and Asia (7, 8).

Both HPAI and LPAI became endemic in land-based poultry in 2007 in Bangladesh (9, 10). Several studies have indicated that migratory birds have played a significant role in the epidemiology and ecology of AIV outbreaks in Bangladesh (4, 11). Wild migratory birds (Mgb) are ubiquitous and highly mobile potential hosts capable of moving pathogens over large distances and across geographical and political borders (1). Bangladesh attracts large numbers of wild birds over winter in wetland habitats. Every year, more than 30 species arrive in the winter season in Bangladesh. Lesser whistling teal, greater whistling teal, cotton pygmy geese, pochard, darters (snakebirds), pintail duck, herons, comb duck, kingfishers, egrets, bitterns, storks, and flycatchers are some of them (4). Bangladesh is said to have approximately 712 species of birds; out of these bird species, approximately 320 are Mgb as the country falls on two major bird migratory flyways—the East Asian–Australasian Flyway and the Central Asian Flyway (4). They often cause only mild infections in birds but can evolve into strains of a highly pathogenic nature causing high mortality in domestic bird populations.

Avian influenza viruses replicate in the trachea and the intestines of infected birds (12), and the oropharynx and cloacae may contain high virus concentrations (13). Feces shed from infected birds also harbor viruses that can maintain infectivity in the environment for long periods (12, 14), especially in the winter season (November to February for Bangladesh). The virus can be transmitted directly between birds or acquired by ingesting contaminated water (15, 16). AIV persists in the environment of wild birds. Environmental persistence may enable short-and long-term maintenance of the virus by providing mechanisms for transmission between spatially or temporally separated bird populations (17), and environmental transmission is crucial for sustaining infection (18). Active AIV surveillance programs utilize live bird capture or hunter-killed birds for AIV testing (19, 20).

Avian coronaviruses (AvCoVs) belong to the gammacoronavirus group and cause infection in poultry and wild birds. Infectious bronchitis is a highly contagious upper respiratory tract disease of chickens (21). The first isolation of infectious bronchitis virus (IBV) was in Massachusetts in the 1930s (22). Gammacoronaviruses have been detected in a range of species of wild birds on all continents (23) and also detected in domestic ducks in Bangladesh. These viruses can affect the upper respiratory tract and the reproductive tract, and some strains can cause nephritis. Morbidity is typically 100%, and mortality is low but can be greater than 50% with some strains that cause nephritis or when opportunistic pathogens such as *Escherichia coli* complicate the disease. In addition, the reproductive tract of layer and breeder birds can be affected, causing decreased egg quality and production (22). For the most part, coronaviruses that affect mammals occur as one or a few different serotypes within a species. In contrast, avian coronavirus (IBV) is characterized by the presence of many different serotypes, making it unique among all other coronaviruses. Gammacoronavirus is a single-stranded positive-sense RNA genome surrounded by a lipid envelope (24). The viral genome encodes several proteins, including the RNA-dependent RNA polymerase (RdRp) and structural proteins such as spike, envelope, membrane, and nucleocapsid proteins. For many years, there was a lack of standardization of IBV strain nomenclature. Recently, most scientists working with IBV have tried to adapt strain designations, but the result is unclear (25). In this study, we conducted AIV and AvCoV surveillance of the environmental samples, mostly feces, collected from migratory bird inhabitants across different areas of three divisions (Dhaka, Sylhet, and Mymensingh) located in central and upper eastern Bangladesh in winter seasons from 2021 to 2024. Since many migratory birds travel from eastern Russia and China to Bangladesh during the winter, this time of year is taken into consideration for sample collection. To evaluate the genetic diversity of the viruses, selective genome sequencing was also carried out on identified AIV subtypes H4N2, H4N6, and AvCoVs.

## 2 Materials and methods

### 2.1 Sample collection

**Figure 1:**
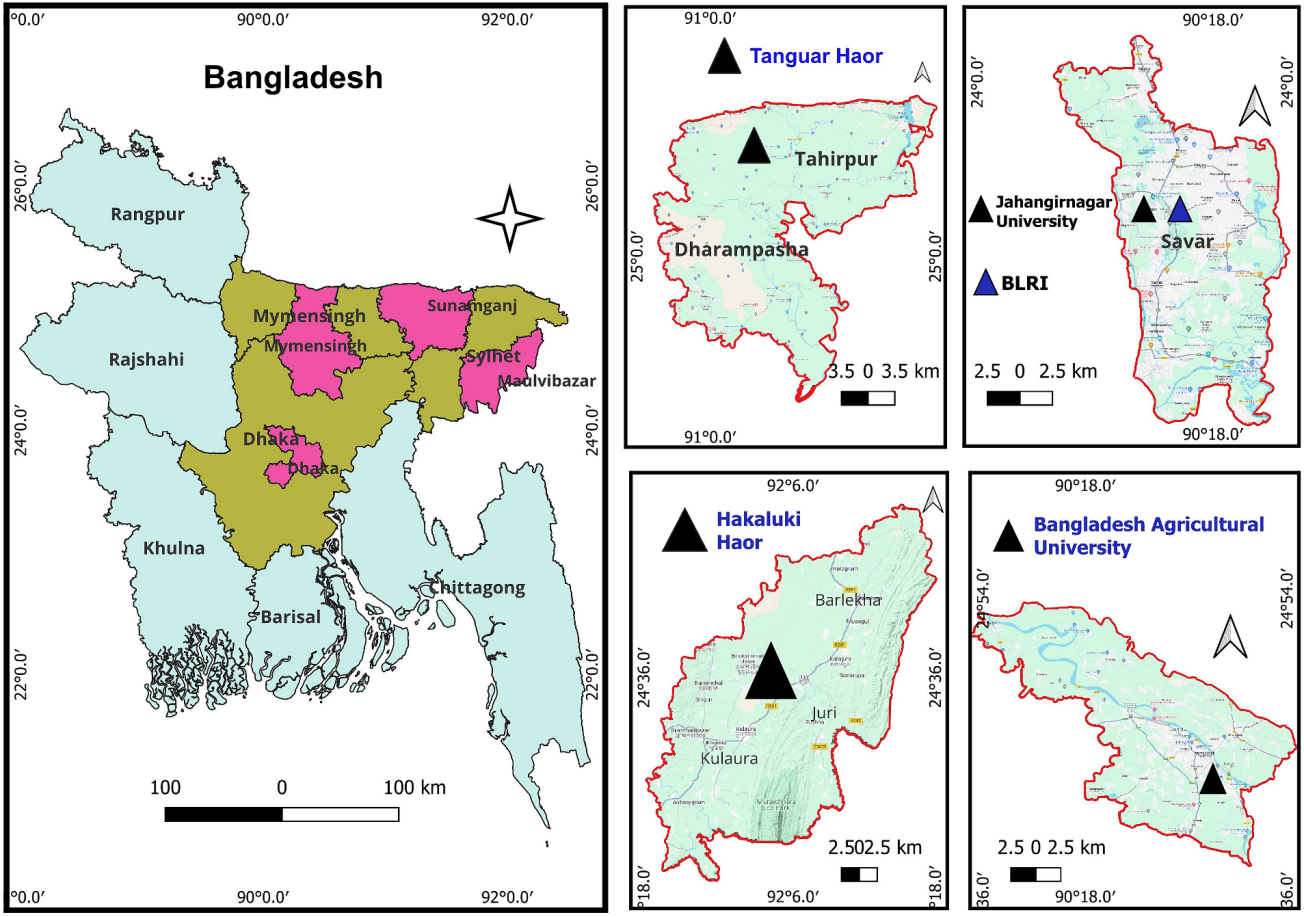
Geographical location of the sampling area from the migratory bird inhabitants taken into consideration for this study. The pink color designates the sample collection area, displayed within each of the three separate divisions (dark brown). The middle and right panels with a triangle symbol (▲) provide a closer view of the areas.

Multiple migratory resident sites at Bangladesh Livestock Research Institute (BLRI) and Jahangirnagar University (JU), in Dhaka division, situated in the central part of the country, at Tanguar haor and Hakaluki haor (haor means wetland ecosystem), respectively, in Sunamganj, Moulavibazar district of Sylhet division located in the eastern part of the country, and at the lakes and riverside of the Brahmaputra in Bangladesh Agricultural University campus at Mymensingh district of Mymensingh division (Figure 1) were monitored for the detection of two major respiratory viral pathogens: avian influenza virus (AIV) and avian coronavirus (AvCoV). The selected areas cover the two major bird migratory flyways—the East Asian–Australasian Flyway and the Central Asian Flyway. The samples were collected over three consecutive winter seasons: 2021–2022, 2022–2023, and 2023–2024. In Bangladesh, the winter season is defined as the months of December, January, and February, during which migratory birds from eastern Russia and China visit the country. Fresh feces from migratory birds on grass blades or along the banks of lakes, haors, and rivers where they spend the night were sampled. The samples were fresh, collected very early in the morning, and had no exposure to the sun. Approximately 1 g of fecal and soil samples were collected using a spoon and placed in a 15-ml sterile Falcon tube containing 2 mL of isotonic solution consisting of phosphate-buffered saline (PBS), pH 7.2, 50% glycerol, penicillin (10,000 U/mL), gentamicin (250 mg/mL), and nystatin (2,500 U/mL). A measure of 10 mL of water from a haor (2 mL from five different sites of each haor is considered one pool) was directly collected and stored in a Falcon tube. Antibiotics were also added, and the probability of obtaining a positive is extremely low considering that the large bodies of water are significantly diluted. The water samples were transferred to a cooling box at 2–8°C until processed in the laboratory. A total of 850 environmental samples were collected. Fecal (*n* = 830), water (*n* = 10), and soil (*n* = 10) samples of three to five droppings or collections from each location in the respective year were pooled and mixed well. The samples were centrifuged at 3,000 rpm for 15 min, and the supernatants were collected. The clarified supernatant was transferred to a new sterile tube and stored at-20°C until RNA extraction.

### 2.2 Molecular screening of AIVs and AvCoVs

The pooled fecal, soil, and water samples (*n* = 184) were processed for nucleic acid extraction. The QIAamp Viral RNA Mini Kit (Qiagen, Hilden, Germany) was used to extract the nucleic acid (RNA) following the kit’s instructions. The samples were screened for detection of AIV and AvCoV following one-step RT-qPCR using TaqMan probe-based and SYBR Green chemistries.

The TaqMan probe-based single-step RT-qPCR assay was optimized to detect the generic AIV M gene, as described previously (1), using an AgPath Universal Probe One-Step RT-qPCR Kit (Thermo Fisher Scientific, United States). The final reaction volume was 12.5 μL, containing 2.5 μL of RNA template of 10–15 ng, 6 μL of 2× RT-PCR reaction mix, 0.5 μL of RT-PCR Enzyme Mix, 1.5 μL of nuclease-free water, and 2 μL of primer-probe mix (10 pmol each). The RT-qPCR thermocycling conditions were set at 45°C for 10 min for reverse transcription, followed by an initial denaturation at 95°C for 10 min. This was followed by 40 cycles of denaturation at 95°C for 15 s and annealing/elongation at 60°C for 1 min, during which time fluorescence was measured. Samples yielding cycle threshold (Ct) values ≤35 were considered positive.

AvCoVs were detected by target primers specific for the untranslated region (5’ UTR, 142 bp) and polymerase gene (Orf 1; 602 bp), respectively, using a single-step RT-qPCR SYBR Green assay. The assay used a Luna Universal One-Step RT-qPCR Kit (New England Biolabs Inc., United States) containing SYBR Green reagents following a previously established protocol (26). Samples yielding cycle threshold (Ct) values ≤35 with an appropriate melting temperature (Tm) were considered positive.

### 2.3 Virus isolation

Virus isolation was conducted on PCR-positive (AIV and AvCoV) individual samples in embryonated chicken eggs using a standard protocol (27). Three consecutive blind passages were performed for each PCR-positive sample. RT-qPCR and hemagglutination assays (HA) were used to confirm the presence of replication-competent viruses in the harvested allantoic fluids.

### 2.4 Sequencing and phylogenetic analysis

Amplification of the full-length hemagglutinin (HA) and neuraminidase (NA) genome segments of AIV and partial polymerase gene fragment of AvCoV was carried out by standard conventional RT-PCR, as described previously (1). Among the PCR positives, the samples that obtained a high concentration of DNA (Ct value ≤25) were considered for sequencing. Selected positive AIV (*n* = 8) and AvCoV (*n* = 6) samples were subjected to commercial Sanger sequencing services. The obtained sequences were subjected to Clustal W multiple sequence alignment and residue analyses using the BioEdit 7.1.5 program. Assembled sequences were then searched in the NCBI database for subtype confirmation. Additional influenza viruses representing H4 HA, N2 NA, N6 NA, and AvCoV polymerase genes available in GenBank and GISAID (Global Initiative on Sharing All Influenza Data) platforms were downloaded for comparative study. Phylogenetic analyses were carried out using the distance-based neighbor-joining (NJ) method with 1,000 bootstrap replicates, using the software MEGA X version. The protein sequences were analyzed for the specific conserved regions in the HA and NA proteins of AIVs. The nucleotide (nt) sequences obtained from this study are available in GISAID (AIVs) and GenBank (AvCoVs) under the accession numbers EPI3358650 to EPI3358665, respectively. The details are shown in Supplementary Table S1.

## 3 Results

### 3.1 Detection of AIVs and AvCoVs

In total, 184 pooled environmental samples collected at sites across the study area were tested, of which 22 were positive for the AIV matrix gene (11.96%) and 15 were positive for the AvCoV combined UTR and polymerase gene (8.15%). The Cq values of AIV-positive samples ranged from 14 to 33, while AvCoV-positive samples had Cq values ranging between 21 and 34 and a melt curve dissociation temperature of 82°C ± 2. The detection summary is shown in Table 1. We did not have permission to catch the migratory bird and collect tissue samples; thus, virus isolation success was limited.

**Table 1:** Comprehensive sampling and detection summary of the avian coronavirus (AvCoV) and avian influenza virus (AIV) obtained from particular regions of Bangladesh.

Division	Sampling area	Year	Sample type	Collected samples (*n*)	Pooled and tested samples (*n*)	Positive samples (*n*)	
						AIV	AvCoV
Dhaka	JU	December 2021	Feces	81	20	6	5
		January 2023	Feces	68	17	1	1
	BLRI	January 2022	Feces	244	49	0	3
		January 2023	Feces	100	26	4	1
Sylhet	Tanguar haor	January 2023	Feces	107	22	4	1
		January 2024	Feces	100	20	6	2
			Soil	10	02	1	0
			Water	10	02	0	0
	Hakaluki haor	February 2024	Feces	100	20	0	0
Mymensingh	BAU	February 2024	Feces	30	06	0	2
Total (feces + soil + water)				850	184	22	15

### 3.2 Area and seasonal prevalence of AIVs and AvCoVs

**Figure 2:**
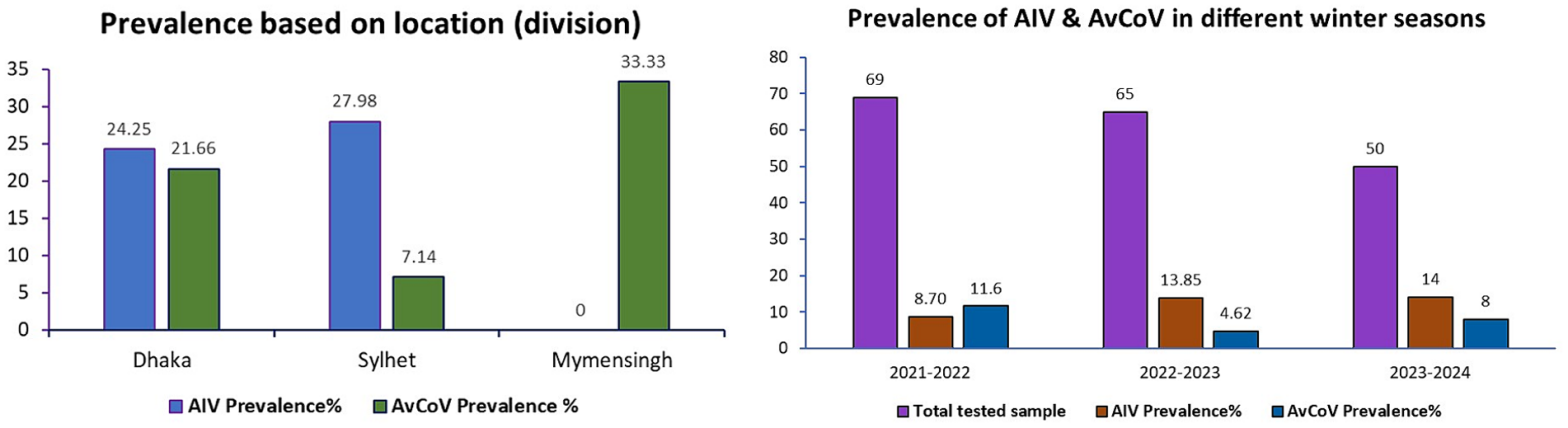
Column graphs depicting the prevalence of AIVs and AvCoVs. The prevalence in the three major divisions chosen as sampling sites for the current study is displayed in the left panel. Three distinct winter seasons are depicted in the right panel. The prevalence was computed using 184 pooled and tested samples.

During the surveillance in three consecutive winter seasons, a total of 37 samples were positive for respiratory viral pathogens either AIVs or AvCoVs, which accounted for 5% of the total number of samples (*n* = 850) and 21% of the total pooled and tested samples (*n* = 185). Out of the pooled and tested samples, 24.25 and 27.98% were found positive for AIVs in the Dhaka and Sylhet divisions, respectively, whereas AIV was negative in samples from the Mymensingh divisions (Figure 2). Comparing the three distinct winter seasons, winter 2023–2024 showed the highest detection rate of AIVs, while winter 2021–2022 showed the highest detection rate of AvCoVs. Nonetheless, the fecal samples of the migratory birds contained two significant viral pathogens (Figure 2).

### 3.3 Subtype confirmation and phylogenetic origin of AIV H4N2 and H4N6

**Figure 3:**
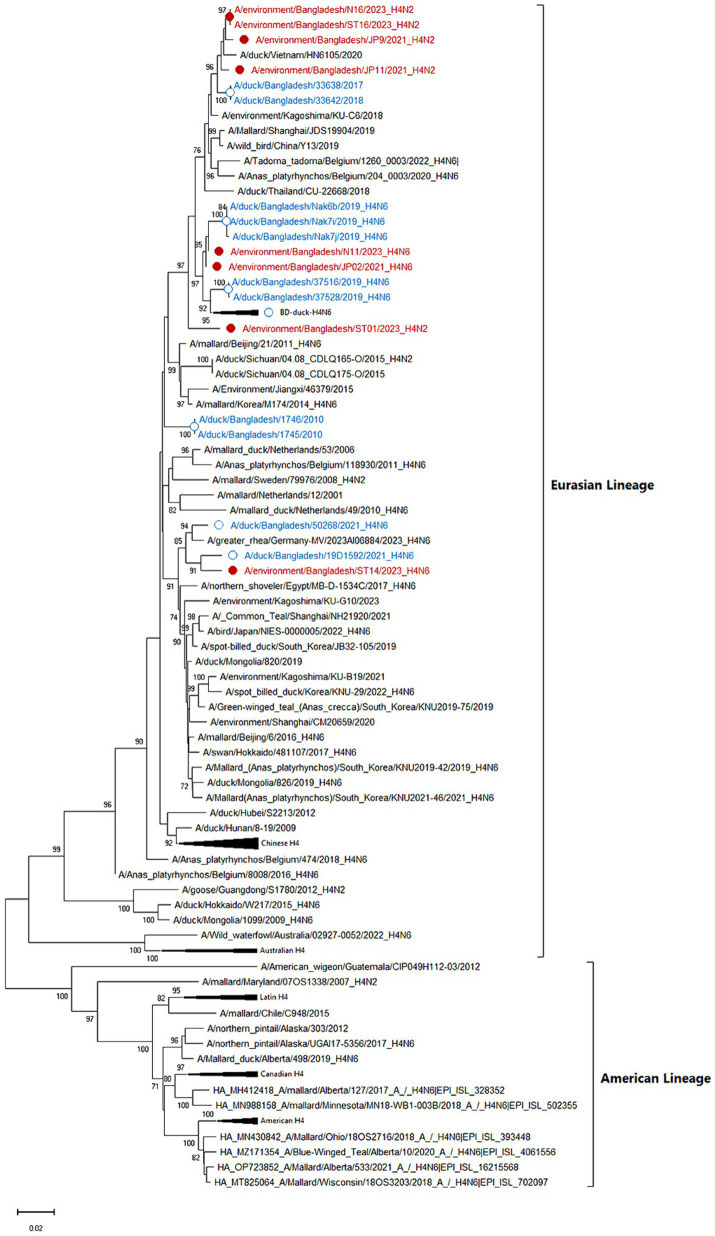
Phylogenetic analysis of HA genes of 101 H4 avian influenza viruses (AIVs). The neighbor-joining (NJ) tree was constructed using MEGA X software. The bootstrap values were calculated on 1,000 replicates, and values less than 70% are not shown. In the figures, red solid circles with red color taxons denote Bangladeshi H4 strains of the current study, while blue hollow circles with blue taxons indicate Bangladesh strains from the previous studies. The tree scale represents the number of substitutions per site.

Among eight sequenced AIVs, five isolates were confirmed as the subtype H4N2, whereas three were confirmed as the subtype H4N6. According to phylogenetic trees, the eight sequenced HA genes of studied AIV strains (JP09, JP12, N16, ST01, ST16, JP02, N11, and ST14) belonged to the Eurasian lineage and were closely related to contemporary strains of Bangladeshi, Chinese, Japanese, Korean, and Vietnamese viruses (Figure 3) with a nucleotide homology of 97.7–99.02%. The Bangladeshi H4 HA is distributed into different subclusters indicating multiple introductions.

**Figure 4:**
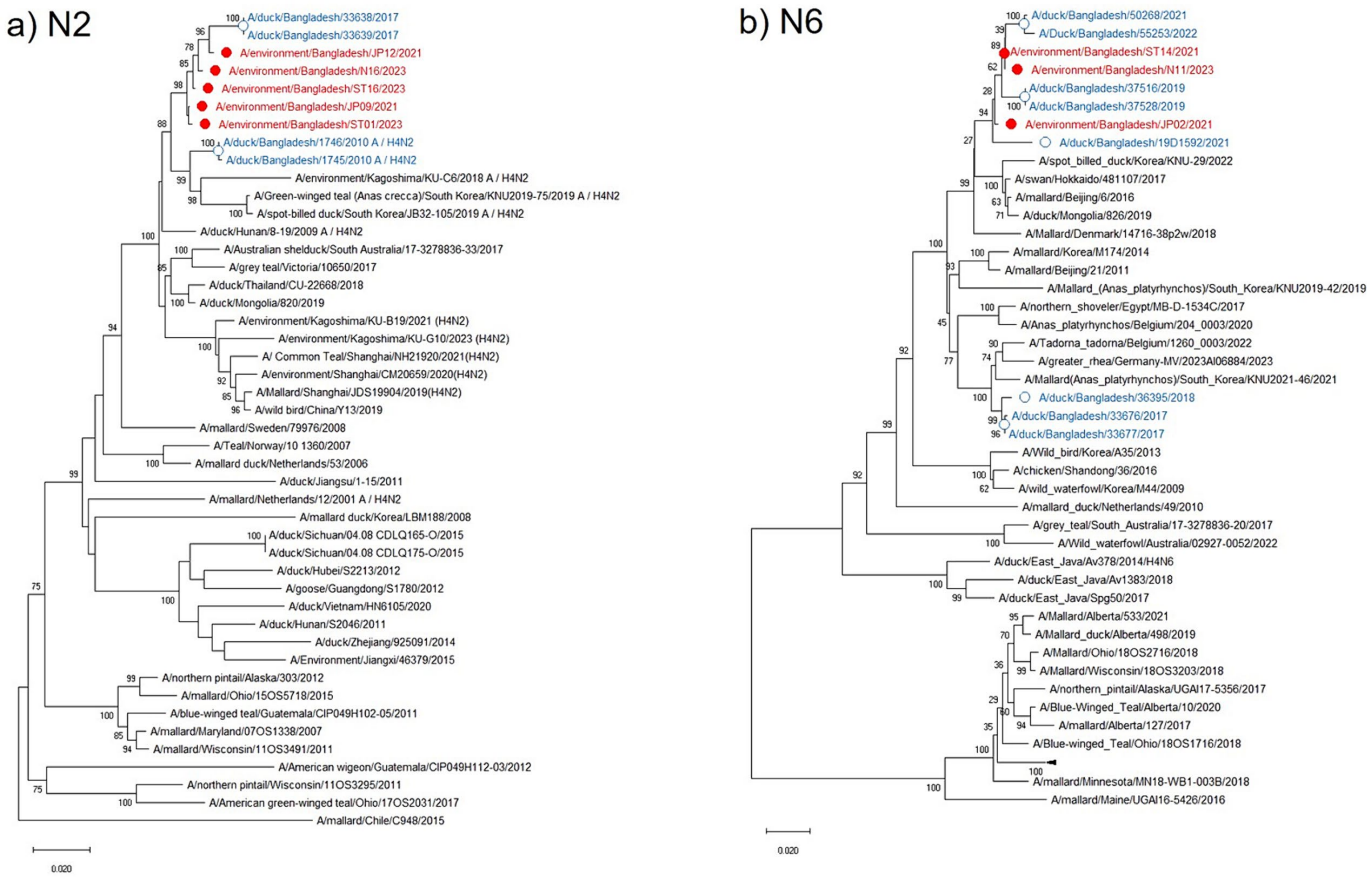
Phylogenetic analysis of NA genes of avian influenza viruses (AIVs). The neighbor-joining (NJ) tree was constructed using MEGA X software. The bootstrap values were calculated on 1,000 replicates, and values less than 70% are not shown. The 46 N2 phylogenetic tree is in the **(a)** panel, and the 50 N6 tree is shown in the **(b)** panel. In the figures, red solid circles with red color taxons denote Bangladeshi H4 strains of the current study, while blue hollow circles with blue taxons indicate Bangladesh strains from the previous studies. The tree scale represents the number of substitutions per site.

The N2 NA phylogenetic tree showed the highest identity (98.91–99.02%) with the Bangladeshi H4N2 subtype isolated in 2017 along with other Asian strains from China, Japan, Korea, Thailand, and Mongolia. It maintained 98.05–98.61% nucleotide identity with the European strains from the Netherlands, Sweden, and Italy (Figure 4a). All current isolates were grouped in a single cluster, and higher nucleotide identity was observed within the H4N2 strains from the past 5 years and clustered with the H4N2 from northeastern Asian strains circulating in ducks and other wild bird populations. Similarly, the phylogenetic tree of the N6 NA in the current study was also clustered in a single group along with previous Bangladeshi isolates and maintained a homologous (98.2–99.6%) association with the isolates from Korea, Japan, China, and Mongolia that were collected from a mallard duck in Denmark (Figure 4b).

All eight H4 IAV strains shared the same amino acid sequence PEKASR/GLF at the HA cleavage site between HA1 and HA2, indicating that all viruses were low-pathogenic influenza viruses. All H4 AIVs had 226Q and 228 G (H3 numbering) at the receptor-binding region, indicating that they maintained the usual avian virus-like receptor specificity (sialic acid-2,3-NeuAcGal).

### 3.4 Phylogenetic origin of AvCoVs

**Figure 5:**
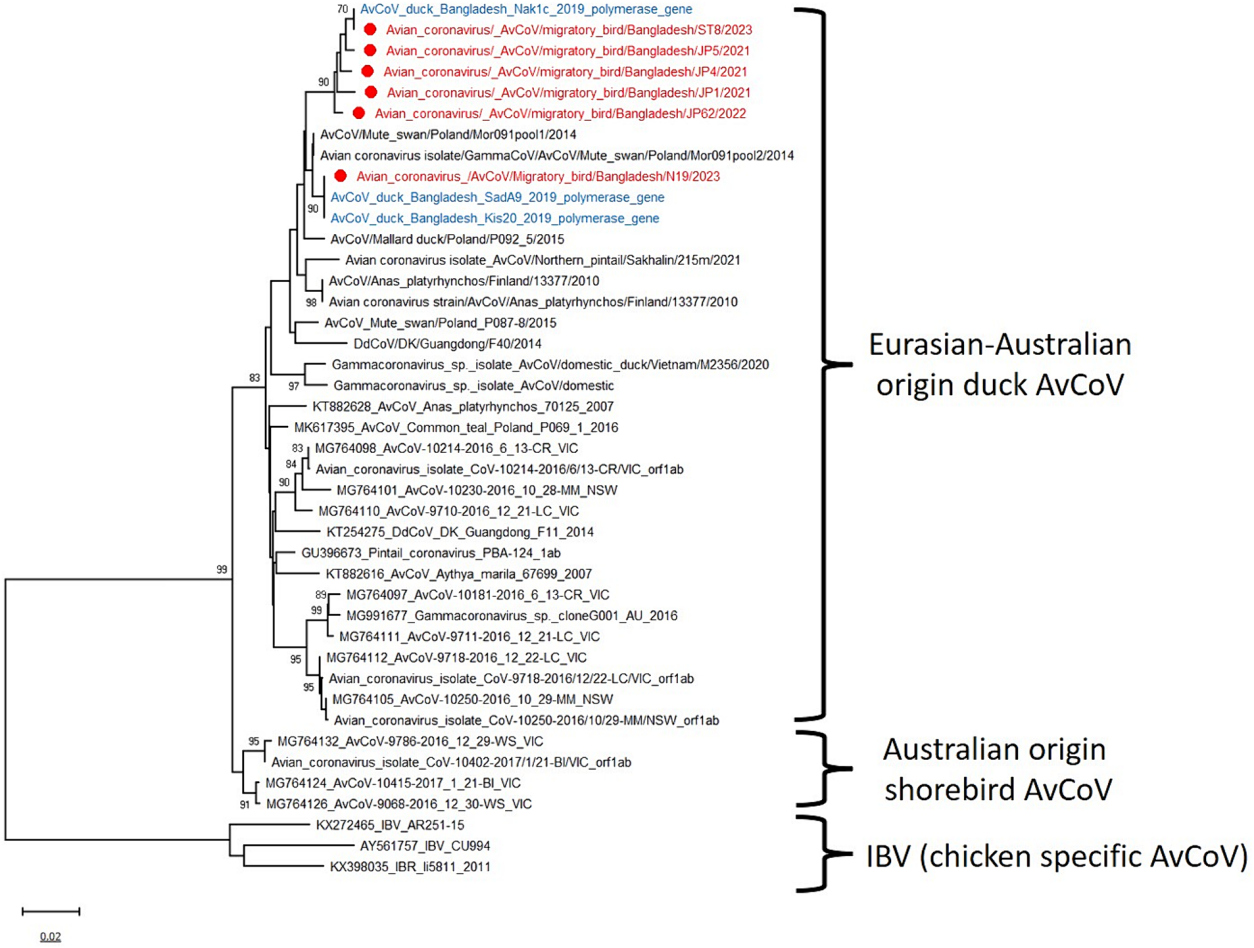
Phylogenetic analysis of partial polymerase genes of 42 avian coronaviruses (AvCoVs). The neighbor-joining (NJ) tree was constructed using MEGA X software. The bootstrap values were calculated on 1,000 replicates, and values less than 70% are not shown. In the figures, red solid circles with red color taxons denote Bangladeshi H4 strains of the current study, while blue hollow circles with blue taxons indicate Bangladesh strains from the previous studies. The tree scale represents the number of substitutions per site.

Among the 15 positive AvCoVs, 6 strains were successfully sequenced. These sequences were closely related to duck AvCoVs of Eurasian and Australian origin (Figure 5). Phylogenetically, AvCoVs are segregated into two main groups: infectious bronchitis virus (IBV)-like AvCoVs and Eurasian-Australian waterfowl AvCoVs, which further branch into Eurasian-Australian-origin duck AvCoVs and Australian-origin shorebird AvCoVs (Figure 5). The previously identified Bangladeshi duck AvCoVs are closely related to the studied AvCoV strains, with a maximum nucleotide identity of 99.4%, indicating that similar viruses are circulating in the country.

## 4 Discussion

The purpose of this study is to monitor the avian influenza viruses (AIVs) and avian coronaviruses (AvCoVs) in migratory bird populations inhabiting lakes, rivers, and other large water bodies such as haors in the winter seasons in Bangladesh. The study area has a high significance for the circulation of AvCoVs and AIVs among migratory birds, and it has been linked to the potential spread of these viruses to nearby waterfowl, primarily ducks (1, 28). The surveillance study conducted over three consecutive winter seasons (2021–2022, 2022–2023, and 2023–2024) analyzed 180 pooled fecal, 2 soil, and 2 water samples (*n* = 850). The birds in the study area were anticipated to be healthy, and no highly pathogenic (H5Nx) avian influenza was detected, although there is an ongoing H5 outbreak in regional poultry in the country (29–32).

In the present study, we detected 22 AIVs and 15 AvCoVs in the environmental samples of these; five were confirmed to be AIV H4N2 subtypes, three to be AIV H4N6 subtypes, and six to be AvCoVs at sequencing. The H4 subtype is frequently isolated in wild birds in many countries, and it is among the most prevalent subtypes in all of Eurasia (6, 33, 34). Phylogenetically, the currently characterized H4 was also clustered with the viruses from eastern Asia and Europe belonging to the Eurasian lineage. Further H4N2 Eurasian lineages were reported previously in the surveillance of live bird market samples in Bangladesh (35, 36) along with wild birds in China, Vietnam, and Korea (34). Phylogenetic analysis of three H4N6 subtypes revealed that they also belonged to the Eurasian lineage and were divided into two clusters. The origin of the Bangladeshi H4N6 suggested that virus introduction may be from the East Asian region (China, Japan, Mongolia, and Korea) via migratory birds (7, 37). Currently studied H4 strains have avian specificity, with no clinical signs, and thus remain unnoticed. However, H4 AIV infection has occurred in humans working with chickens (38, 39), indicating that H4 has the potential to cross species barriers to infect humans. During their replication in mammals, H4 AIVs may acquire new mutations and become more virulent or easily transmissible (40). Acquiring efficient respiratory droplet transmission among humans is a prerequisite for the outbreak of an influenza pandemic. According to a recent report, H4 viruses could be transmitted to guinea pigs via direct contact and respiratory droplets (40).

Wild migratory birds are natural reservoirs of IAVs, genetically found as low pathogenic, but viruses evolve through mutation and reassortment (41). Bangladesh is geographically significant because it is situated along two major migratory flyways, which combine to provide an ideal winter habitat for wild migratory birds. Moreover, the country has experienced ongoing outbreaks of highly pathogenic avian influenza subtype H5N1 and low-pathogenic avian influenza subtype H9N2 in commercial poultry and live bird markets throughout the year (26, 28, 31). Therefore, the presence of AIVs H4N2 and H4N6 in the migratory bird inhabitants poses a potential threat to domestic waterfowl to transmit and spread the disease further into other land-based poultry.

Avian coronaviruses (AvCoVs) were detected in 8.15% of pooled fecal samples (*n* = 184) and detected in all three divisions under surveillance. The polymerase (orf1ab polyprotein) gene sequence of six positive AvCoVs confirmed the circulation of gammacoronaviruses (gamma-CoV) in migratory bird inhabitants. Infectious bronchitis virus (IBV), another member of the gamma-CoV genus, is the most significant disease prevalent among chickens in Bangladesh (42). However, the detected AvCoVs belong to the Eurasian-Australian-origin duck AvCoV group, distinct from IBV-like AvCoV (Figure 5). The spike (S) gene sequencing could provide a summary of the genotypes that are being studied. Australian-origin shorebird AvCoVs made a separate cluster, and such clustering by host species or order was observed in coronavirus sequences in Australia, Hong Kong, China, and Cambodia (43–45). Birds of both orders, Charadriiformes and Anseriformes, are susceptible to coronaviruses, and regular spill-over transmission and geographical spread of the AvCoV have been described (44). The circulation of AvCoVs in Bangladeshi domestic ducks and migratory bird inhabitants can act as a source and can be attributed to direct or indirect contact with infected migratory waterfowl moving along the East Asian–Australasian Flyway (45).

## 5 Conclusion

To summarize, this study describes that AIV H4N2, H4N6, and AvCoVs are detected in the migratory bird population in mid-eastern Bangladesh during winter months and are potentially significant avian viral pathogens. Active surveillance in migratory bird habitats helps in the spread of the viruses to domestic poultry to commercial poultry holdings and trading networks via domestic waterfowl, which may help better understand the dispersal and transmission dynamics of the viruses. Furthermore, it may contribute to understanding the diversity, distribution, and circulation of coronaviruses and avian influenza.

## Data Availability

The datasets presented in this study can be found in online repositories. The names of the repository/repositories and accession number(s) can be found in the article/Supplementary material.
