# Supplementary material for: Molecular epidemiology of avian influenza viruses and avian coronaviruses in environmental samples from migratory bird inhabitants in Bangladesh
Source: Front Vet Sci. 2024 Oct 7;11:1446577. doi: 10.3389/fvets.2024.1446577 (PMC11491338; doi:10.3389/fvets.2024.1446577)
Supplement: Supplementary file 1 [file Table_1.DOCX]

Supplement Table S1: Accession number of submitted sequence of migratory bird samples to the publicly accessed platform Global Initiative on Sharing All Influenza Data (GISAID).

| **Strain name** | **Subtype** | **Gene** | **Accession Number (GISAID)** |
| --- | --- | --- | --- |
| JP02 | H4N6 | HA | EPI3358650 |
| JP02 |  | NA | EPI3358651 |
| N11 | H4N6 | HA | EPI3358652 |
| N11 |  | NA | EPI3358653 |
| ST14 | H4N6 | HA | EPI3358654 |
| ST14 |  | NA | EPI3358655 |
| JP9 | H4N2 | HA | EPI3358656 |
| JP9 |  | NA | EPI3358657 |
| JP12 | H4N2 | HA | EPI3358658 |
| JP12 |  | NA | EPI3358659 |
| N16 | H4N2 | HA | EPI3358660 |
| N16 |  | NA | EPI3358661 |
| ST01 | H4N2 | HA | EPI3358662 |
| ST01 |  | NA | EPI3358663 |
| ST16 | H4N2 | HA | EPI3358664 |
| ST16 |  | NA | EPI3358665 |
